# Liraglutide preserves CD34^+^ stem cells from dysfunction Induced by high glucose exposure

**DOI:** 10.1186/s12933-022-01486-9

**Published:** 2022-04-09

**Authors:** Annalisa Sforza, Vera Vigorelli, Erica Rurali, Gianluca Lorenzo Perrucci, Elisa Gambini, Martina Arici, Alessia Metallo, Raffaella Rinaldi, Paolo Fiorina, Andrea Barbuti, Angela Raucci, Elena Sacco, Marcella Rocchetti, Giulio Pompilio, Stefano Genovese, Maria Cristina Vinci

**Affiliations:** 1 Unit of Vascular Biology and Regenerative Medicine, Centro Cardiologico Monzino IRCCS, Via C. Parea 4, 20138 Milan, Italy; 2 Department of Biotechnology and Biosciences, Università degli Studi di Milano-Bicocca, Milan, Italy; 3 Division of Endocrinology, ASST Fatebenefratelli-Sacco, Milan, Italy; 4 International Center for T1D, Pediatric Clinical Research Center Romeo ed Enrica Invernizzi, DIBIC, Università di Milano, Milan, Italy; 5 Nephrology Division, Boston Children’s Hospital, Harvard Medical School, Boston, MA USA; 6 Department of Biosciences, Università degli Studi di Milano, Milan, Italy; 7 Unit of Experimental Cardio-Oncology and Cardiovascular Aging, Centro Cardiologico Monzino IRCCS, Milan, Italy; 8 Department of Biomedical, Surgical and Dental Sciences, Università degli Studi di Milano, Milan, Italy; 9 Diabetes, Endocrine and Metabolic Diseases Unit, Centro Cardiologico Monzino IRCCS, Milan, Italy

**Keywords:** GLP-1 receptor agonist, CD34^+^ hematopoietic stem progenitor cells, Type 2 diabetes mellitus, Cardiovascular disease

## Abstract

**Background:**

Glucagon like peptide-1 receptor agonists (GLP-1RAs) have shown to reduce mortality and cardiovascular events in patients with type 2 diabetes mellitus (T2DM). Since the impairment in number and function of vasculotrophic circulating CD34^+^ hematopoietic stem progenitor cells (HSPCs) in T2D has been reported to increase cardiovascular (CV) risk, we hypothesized that one of the mechanisms whereby GLP-1 RAs exert CV protective effects may be related to the ability to improve CD34^+^ HSPC function.

**Methods:**

In cord blood (CB)-derived CD34^+^ HSPC, the expression of GLP-1 receptor (GLP-1R) mRNA, receptor protein and intracellular signaling was evaluated by RT-qPCR and Western Blot respectively. CD34^+^ HSPCs were exposed to high glucose (HG) condition and GLP-1RA liraglutide (LIRA) was added before as well as after functional impairment. Proliferation, CXCR4/SDF-1α axis activity and intracellular ROS production of CD34^+^ HSPC were evaluated.

**Results:**

CD34^+^ HSPCs express GLP-1R at transcriptional and protein level. LIRA treatment prevented and rescued HSPC proliferation, CXCR4/SDF-1α axis activity and metabolic imbalance from HG-induced impairment. LIRA stimulation promoted intracellular cAMP accumulation as well as ERK1/2 and AKT signaling activation. The selective GLP-1R antagonist exendin (9–39) abrogated LIRA-dependent ERK1/2 and AKT phosphorylation along with the related protective effects.

**Conclusion:**

We provided the first evidence that CD34^+^ HSPC express GLP-1R and that LIRA can favorably impact on cell dysfunction due to HG exposure. These findings open new perspectives on the favorable CV effects of GLP-1 RAs in T2DM patients.

**Supplementary Information:**

The online version contains supplementary material available at 10.1186/s12933-022-01486-9.

## Background

Type 2 diabetes mellitus (T2DM) has now attained the status of a global pandemic with over 400 million individuals affected worldwide [1]. Despite glucose lowering therapies, mortality from cardiovascular disease (CVD) remains high and extremely costly for health care systems both in terms of medical expenses and disability-adjusted life years [2]. For these reasons, the development of new therapeutic strategies able to prevent CVD morbidity and mortality is crucial. Patients with T2DM are characterized by a significant decrease in circulating CD34^+^ stem/progenitor cells. CD34^+^ hematopoietic stem/progenitor cells (HSPCs) are known to possess vascular regenerative and proangiogenic capacity [3]. Their functional and numerical depletion is now considered a significant contributor to CV homeostasis impairment in diabetes. To this regard, Fadini et al*.* demonstrated that CD34^+^ HSPCs are reduced of about 40% in T2DM [4], and that such impairment contributes to enhanced CV risk [5]. Notably, in patients with T2DM, the reduction of CD34^+^ HSPCs number and function predicts adverse CV outcomes, defined as major CV events (MACE), and hospitalizations for CV causes [6, 7]. Recent large-scale trials have unequivocally demonstrated the ability of glucagon-like peptide 1 receptor agonists (GLP1-RAs) to reduce the risk of MACE in T2DM patients with established or at high risk of CVD [8–10]. GLP1-RAs are now recommended by guidelines as first-line agent for prevention of CVD in T2DM patients [11, 12]. Such pleiotropic CV benefit appears to be additional to glucose-lowering effects and the mechanisms whereby they exert such striking CV protective effects are still largely unknown. At cellular and molecular level, GLP1-RA effects are mediated by GLP1-R, a Gs coupled receptor family member, which is present in various human tissues [13]. To date, there are no data describing the effects of GLP1-RAs on CD34^+^ HSPCs of T2DM patients. We hypothesized that at least part of the unknown mechanisms whereby GLP1-RAs exert CV protective effect are mediated by its ability to improve CD34^+^ HSPC function. Here, by exploiting an in vitro model of diabetes, we show for the first time that CD34^+^ HSPCs express GLP-1R and that its stimulation by liraglutide (LIRA), a GLP1-RA, prevents and recovers the dysfunction induced by hyperglycemia.

## Methods

### Experimental design

**Fig. 1:**
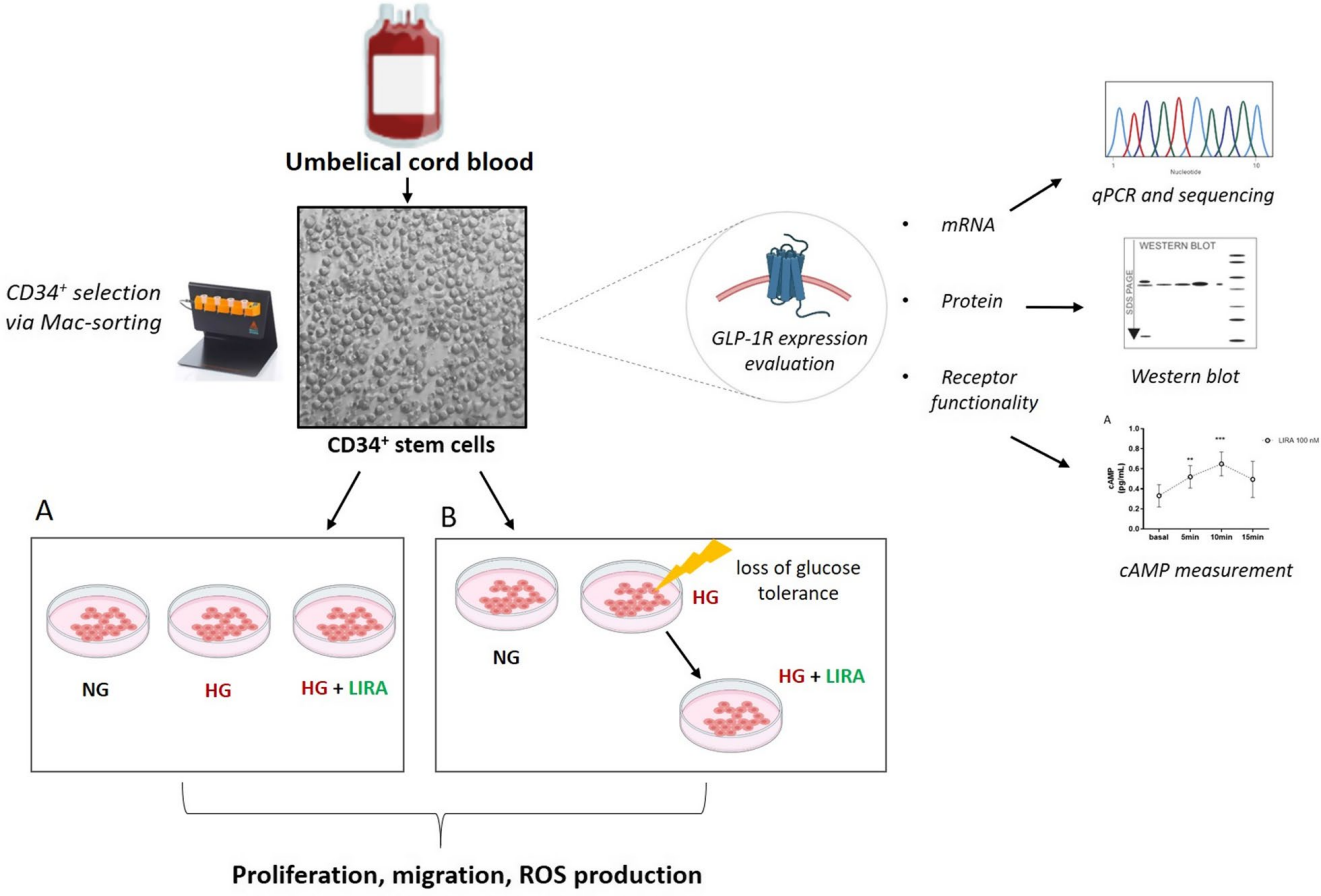
Schematic representation of the study. CD34^+^ HSPCs were isolated via Mac-sorting from cord blood and characterized for GLP-1R transcript and protein expression levels, and downstream signaling pathway activation. Then, CD34^+^ HSPCs were expanded in HG condition with or without LIRA (**A**). In a different experimental setting the cells were treated with LIRA only after the loss of glucose tolerance (**B**). CD34^+^ HSPCs cultured in NG (30 mM mannitol) condition were used as control (**A**, **B**). Proliferation, migration and oxidative stress were finally assessed at the end of experiments. GLP-1R = glucagone-like peptide 1 receptor; HG = high glucose; LIRA = liraglutide; NG = normal glucose

We recently established a stem cell culture model of diabetes based on the use of cord blood (CB)-derived CD34^+^ HSPCs [14]. This method already provided a consistent and reproducible recapitulation of the major CD34^+^ HSPC dysfunction hallmarks in diabetes [14]. To assess the ability of GLP-1 RA to prevent CD34^+^ HSPC dysfunction induced by glucose overload the cells were expanded in high glucose (HG; 30 mM) conditions along with 50 nM or 100 nM LIRA treatment (Fig. 1A). In a different experimental setting, CD34^+^ HSPCs were expanded in HG condition and then treated with LIRA only after loss of glucose tolerance (Fig. 1B). Afterwards, we assessed the ability of the drug to recover a compromised phenotype. CD34^+^ HSPCs cultured in normoglycemic condition (NG; 30 mM mannitol) were used as control. At the end of both experiments, the main dysfunctional hallmarks of the cells, namely proliferation and CXCR4/SDF-1α axis impairment, were evaluated (Fig. 1).

### Cell culture

Umbilical cord blood (UCB) was collected from the umbilical cord of full-term normal deliveries in collaboration with Milano Cord Blood Bank (IRCSS Ca’ Granda Foundation – Ospedale Maggiore Policlinico). The mononuclear cell fraction was obtained by density gradient centrifugation using Ficoll-Paque (Lymphoprep, Sentinel Diagnostics) and CD34^+^ HSPCs were immunomagnetically isolated using CD34 Microbead Kit (MiniMACS kit, Miltenyi Biotec). Flow-cytometric analysis allowed to assess the purity of sorted cell population, displaying 90% of CD34^+^ HSPCs and negligible presence of CD14^+^ (monocytes) and CD3^+^ (lymphocytes) cells (Additional file 1: Figure S1). Isolated CD34^+^ HSPCs were cultured in Stem Span medium (StemCell Technologies) supplemented with 20 ng/mL of interleukin (IL)‐6 (PeproTech), 20 ng/mL of IL‐3 (PeproTech), 50 ng/mL of fms‐like tyrosine kinase 3 (FLT3, PeproTech), and 50 ng/mL of stem cell factor (SCF, PeproTech). Cells were cultured in HG (30 mM of glucose, Sigma-Aldrich) or NG (30 mM of mannitol, Sigma-Aldrich) conditions for up to 20 days and treated or not with increasing concentration of LIRA (50 nM and 100 nM; MedCHemExpress) ± selective GLP-1R antagonist exendin (9-39) (150 nM EXE; MedCHemExpress).

Capan-1 cells (HTB-79^™^), used as GLP-1R positive control, were purchased from ATCC and grown in RPMI medium supplemented with 20% FBS, as indicated by the supplier.

### *Isolation of CD34*^+^*HSPCs from patient bone marrow*

CD34^+^ HSPCs were isolated from sternal bone marrow (BM) biopsy of T2DM patients underwent bypass surgery. During surgical procedure, 2 mL of sternal BM blood were withdrawn by biopsy needle (15G × 25/90 mm; MDL) and suspended in saline buffer solution. BM-derived CD34^+^ HSPCs were isolated as aforementioned and collected for GLP-1R mRNA analysis.

All experiments were carried out upon approval of local ethic committees (CCM 205–RE 3428) and informed written consent was obtained from all patients before BM harvesting.

### Cell proliferation assay

CD34^+^ HSPCs were seeded at an initial density of 2.0 × 10^5^ cells/well and cultured for up to 20 days in NG and HG ± LIRA conditions. Cells were counted on days 5, 10, 15 and 20. Doubling time was calculated with the following formula:

$${\text{Doubling}}\,{\text{time: }}\frac{duration\,\;x\,\;\log \,\left( 2 \right)}{{\log \;\,\left( {final\,concentration} \right) - \log \,\;\left( {initial\,concentration} \right)}}$$

### Migration assays

Cell migration was determined the use of Boyden modified chamber consisting of transwell culture inserts (5‐μm pore membrane; Corning Incorporated, Corning, NY). In brief, 1 × 10^5^ cells were seeded onto the upper chamber and allowed to migrate toward the lower chamber containing, or not, stromal cell-derived factor 1 (SDF‐1α 50 ng/mL; PeproTech EC Ltd.). The transwells were incubated at 37 °C, 5% CO_2_, for 4 h. Migrated cells in the lower chamber were counted and migration index were calculated with the following formula:

$${\text{Migration}}\,{\text{index: }}\frac{migrated\,cells\,\,in\,\,presence\,\,of\,SDF - 1a}{{migrated\,cells\,\,in\,\,absence\,\,of\,SDF - 1a}}$$

### Cyclic adenosine monophosphate (cAMP) quantification

Intracellular cAMP was quantified by cAMP ELISA kit (Enzo Life Science) according to manufacturer’s instructions. Briefly, 5 × 10^5^ CD34^+^ HSPCs were stimulated with LIRA ± EXE and lysed in 0.1 M HCl and 0,1% Triton X-100. Sample absorbance was spectrophotometrically evaluated at 405 nm by Tecan (Infinite M200 Pro, TECAN).

### *Intracellular Ca*^*2*+^*handling*

The measurement of intracellular Ca^2+^ has been assessed through single cell and population analysis by means of confocal Nikon A1R microscope and FLUOstar Omega (BMG Labtech) multiplate reader respectively. CD34^+^ HSPCs were starved for 2 h (with IMDM and albumin 0.1%) and incubated with the Ca^2+−^sensitive dye Fluo-4 AM (ThermoFisher, 2 µM) in Tyrode’s solution (containing in mM: 154 NaCl, 4 KCl, 2 CaCl2, 1 MgCl2, 5 HEPES/NaOH, and 5.5 d-glucose, adjusted to pH 7.35) for 1 h.

Confocal single cell analysis was performed by plating CD34^+^ HSPCs on fibronectin/polylisin D coated glass coverslips; fluorescence (F) images (512*512 pxls) were acquired at × 60 magnification every 5 s in basal condition and following 100 nM LIRA addition at 37 °C and 5% CO2 thanks to Okolab incubator mounted on the microscope stage. Changes in single cell mean F during acquisition time were quantified through NIS-Elements analysis software following F backgroud subtraction. Population analysis was performed by plating cells in 96-well dark plates by means of the multiplate reader equipped with an automatic injection system to inject LIRA (100 nM). F was acquired in each well every 0.74 s for 20 s just prior to compound injection and for 100 s after injection. Mean F prior compound injection was used as reference (F0) for signal normalization (F/F0).

### RNA extraction and RT-qPCR

Total RNA from both cord-blood and sternal CD34^+^ HSPCs was isolated by using the Direct‐zol RNA Kit (Zymo Research), following manufacturer’s protocol. One µg of total RNA was converted to cDNA with the Superscript III kit (Life Technologies) and used to assess GLP-1R gene expression. qPCR reactions were performed with SYBR Green Supermix 2X (BIO‐RAD Laboratories) on CFX96 Real–Time System PCR (BIO‐RAD Laboratories). Specific GLP-1R primers (Fw: 5'-GTGTGGCGGCCAATTACTAC-3'; Rv: 5'-CTTGGCAAGTCTGCATTTGA-3') were appositely designed to evaluated mRNA expression by amplifying a region of 347 bp. The qPCR products were loaded on a 1% agarose gel with an appropriate molecular marker (PCR Marker Solution, Sigma-Aldrich). Then, the 347 bp bands were excised and purified with QIAquick Gel extraction kit (Qiagen) for subsequent Sanger sequencing analysis.

### Sanger sequencing

The RT-qPCR products, appropriately purified from agarose, were sequenced with the help of an external service (Microsynth Biotech) by Sanger method with the use of GLP-1R Fw primer. Sequencing results were analysed by a Multiple sequence ClustalW alignment (BioEdit software) throughout the comparison of the published GLP-1R cDNA sequence (NCBI Reference sequence: NM_002062.5).

### Western blot

CD34^+^ HSPCs and Capan-1 cells were lysed in lysis buffer (50 mM TRIS-HCl, 150 mM NaCl, 1 mM EDTA, 1% Triton) added with protease inhibitors (1:10, Halt Protease Inhibitor Cocktail, Thermo Scientific). Protein lysate was then quantified by Pierce™ BCA Protein Assay Kit (ThermoFisher Scientific). Fourty μg and 20 μg of protein from CD34^+^ stem cells and capan-1 cells respectively were resolved on 10% SDS‐PAGE in denaturing conditions. Proteins were then transferred onto a polyvinylidene difluoride (PVDF) membrane (Millipore) at 400 mA, 4 °C for 90 min. To prevent aspecific binding, the membrane was blocked with 5% bovin serum albumin (BSA) in PBS + 0, 1% Tween-20 (PBST) for 1 h. The membranes were then incubated with the primary antibodies, appropriately diluted in 3% BSA-PBST, at 4 °C O/N and with the appropriate secondary antibody linked to horseradish peroxidase (HRP) the day after for 1 h. Specific information about antibodies and appropriate dilutions are reported in Table 1. The signal was detected by Enhanced chemiluminescence (ECL) system and quantified by Chemidoc MP Imaging System (BIO-RAD Laboratories). GLP-1R-mediated AKT and ERK1/2 pathway activation was evaluated by treating the cells with 100 nM LIRA ± 1 µM wortmannin (Sigma Aldrich; WT; inhibitor of phosphatidylinositol 3-kinase, PI3K), 100 µM PD 98059 (Sigma Aldrich; PD; a specific inhibitor of mitogen-activated protein kinase kinase 1/2, MEK1/2), or 150 nM EXE (selective GLP-1R antagonist) and with either no additions as control.

**Table 1:** List of Western blot antibodies

	Code (purchased from)	Source	Dilution
Primary antibody			
GLP-1R (D-6)	Sc-390774 (Santa Cruz Biotecnology)	Mouse	1:200
Phospho-p44/42 MAPK (ERK1/2) (Thr202/Tyr204)	#4370 (Cell signaling)	Rabbit	1:2000
p44/42 MAPK (ERK1/2)	#9102 (Cell signaling)	Rabbit	1:1000
Phospho-AKT (Ser473)	#9271 (Cell signaling)	Rabbit	1:1000
Secondary antibody			
ECL Anti-rabbit IgG	NA9340 (Amersham Biosciences)	donkey	1:10000
ECL Anti-mouse IgG	NA9310 (Amersham Biosciences)	sheep	1:5000

### Flow cytometric assays

CD34^+^ HSPCs were incubated for 30 min with allophycocyanin‐conjugated monoclonal antihuman CXCR4 antibody (BD Biosciences) or with CellROX Green Flow Cytometry Assay Kit (Life Technologies) for the detection of CXCR4 and reactive oxygen species (ROS) respectively. The Gallios Flow Cytometer platform (Beckman Coulter Life Sciences) was used to analyze the samples after appropriate physical gating. At least 20^4^ events in the indicated gates were acquired.

### Immunocytochemistry

CD34^+^ HSPCs were temporarily adhered to a glass coverslip surface by mixed fibronectin-polylysine D (1:1) coating solution. Immediately after adhesion, cells were incubated with Green CellROX (Life Technologies) for 20 min and then fixed with 2% paraformaldehyde solution. Before mounting, cells were counterstained with Hoechst for the nuclei (1:1000) and Wheat Germ Agglutinin for the cell membrane (WGA) (1:200). The images were acquired by ZEISS Apotome fluorescence microscope at 40X magnification.

### Analysis of mitochondrial and glycolytic bioenergetic parameters

Bioenergetic parameters were analyzed by using Seahorse XFe96 Extracellular Flux analyzer (Agilent). Before the analysis, cells were collected and counted: 35 × 10^3^ cells were suspended in 50 µL low buffered DMEM-based XF assay medium (103575-100 Agilent) supplemented with 10 mM glucose, 2 mM glutamine, 1 mM Na-pyruvate, and plated in a fibronectin-polylysine D coated 96-well XF plate (Agilent). The XF plate was centrifuged at 200 g (zero braking) for 1 min and incubated for 20 min at 37 °C in a no-CO_2_ incubator. Before testing, 150 µL complete XF assay medium was added to each well and cells were furtherly incubated for 30 min at 37 °C.

Mitochondrial bioenergetic parameters were analyzed according to the Seahorse Mito Stress test kit protocol (Agilent) that include oxygen consumption rate (OCR; pmolO_2_/min) measurements under basal condition and after the sequential injection of the ATP synthase inhibitor oligomycin A (1.5 µM), the ETC accelerator ionophore FCCP (carbonilcyanide p-triflouromethoxyphenylhydrazone, 2 µM), and the ETC inhibitors mixture rotenone (0.5 µM) + antimycin A (0.5 µM). The minimal doses of oligomycin A and FCCP causing the maximal response used in the Mito Stress test assay were determined for each experimental group in a preliminary Seahorse assay (Additional file 2: Figure S2).

Glycolytic bioenergetic parameters were analyzed by combining the Seahorse ATP rate and Glyco rate test kit protocols (Agilent) including extracellular acidification rate (ECAR; mpH/min) measurements under basal condition and after the sequential injection of 1.5 µM oligomycin A, 0.5 µM rotenone + 0.5 µM antimycin A mixture, and finally 50 mM 2-Deoxy-D-Glucose (2DG).

Seahorse parameters were normalized to cell number in each well. To this purpose, at the end of the Seahorse analysis, cell nuclei were marked with Hoechst 33342 (1 µg/mL) for about 15 min and acquired with Leica Thunder Imager microscope through a 10x objective. Cell number was quantified through ImageJ software. Samples were analyzed with at least 10 technical replicates from two independent experiments. Bioenergetic parameters were calculated from the Seahorse data using Wave2.6.1 (Agilent) software.

Respiratory parameters were calculated using the following formulas:

Basal Mitochondrial Respiration (Basal MR) = OCR under basal condition (OCR_basal_)–OCR following rotenone/antimycin A injection (OCR_rot/ant_); Maximal MR (Max MR) = OCR following FCCP injection (OCR_FCCP_) − OCR_rot/ant_; Spare respiratory capacity = Max MR–Basal MR; Coupling efficiency = delta OCR following oligomycin A injection (OCR_basal_-OCR_oligo_)/OCR_basal_; Proton Leak = OCR_oligo_-OCR_rot/ant_.

Glycolytic parameters were calculated using the following formulas:

Proton Efflux rate (PER; pmolesH^+^/min) was calculated from Extracellular Acidification Rate (ECAR) applying the Buffer Factor of the XF assay medium; Basal glycolysis = PER under basal condition (PER_basal_)–PER following 2DG injection (PER_2DG_); Maximal glycolysis = PER following rotenone/antimycin A injection (PER_rot/ant_)–PER_2DG_.

ATP rate parameters were calculated using the following formulas:

ATP linked respiration (OCR_ATP_) = OCR_basal_–OCR_oligo_; mitoATP production rate = OCR_ATP_ *2 * P/O (P/O = 2.75); mitoOCR = OCR_basal_–OCR_rot/ant_; mitoPER = mitoOCR * CO_2_ Contribution Factor (CCF = 0.61); PER = ECAR * Buffer Factor * VolXF microchamber * Kvol (Kvol = 1.6); glycoATP production rate (glycoPER) = PER–mitoPER.

### Statistical analysis

Results are given as mean ± SEM. All experiments were performed at least in triplicate, unless stated otherwise. The data were tested for the normality by using the Shapiro–Wilk normality test. Differences between data were evaluated by 1‐way, 2‐way repeated‐measures ANOVA followed by the post‐hoc Newman–Keuls or Tukey’s multiple comparison test, as appropriate. A value of P ≤ 0.05 was considered significant. All statistical analysis was performed using GraphPad Prism software (GraphPad Software Inc.).

## Results

### *CD34*^+^*HSPCs express GLP-1 receptor*

**Fig. 2:**
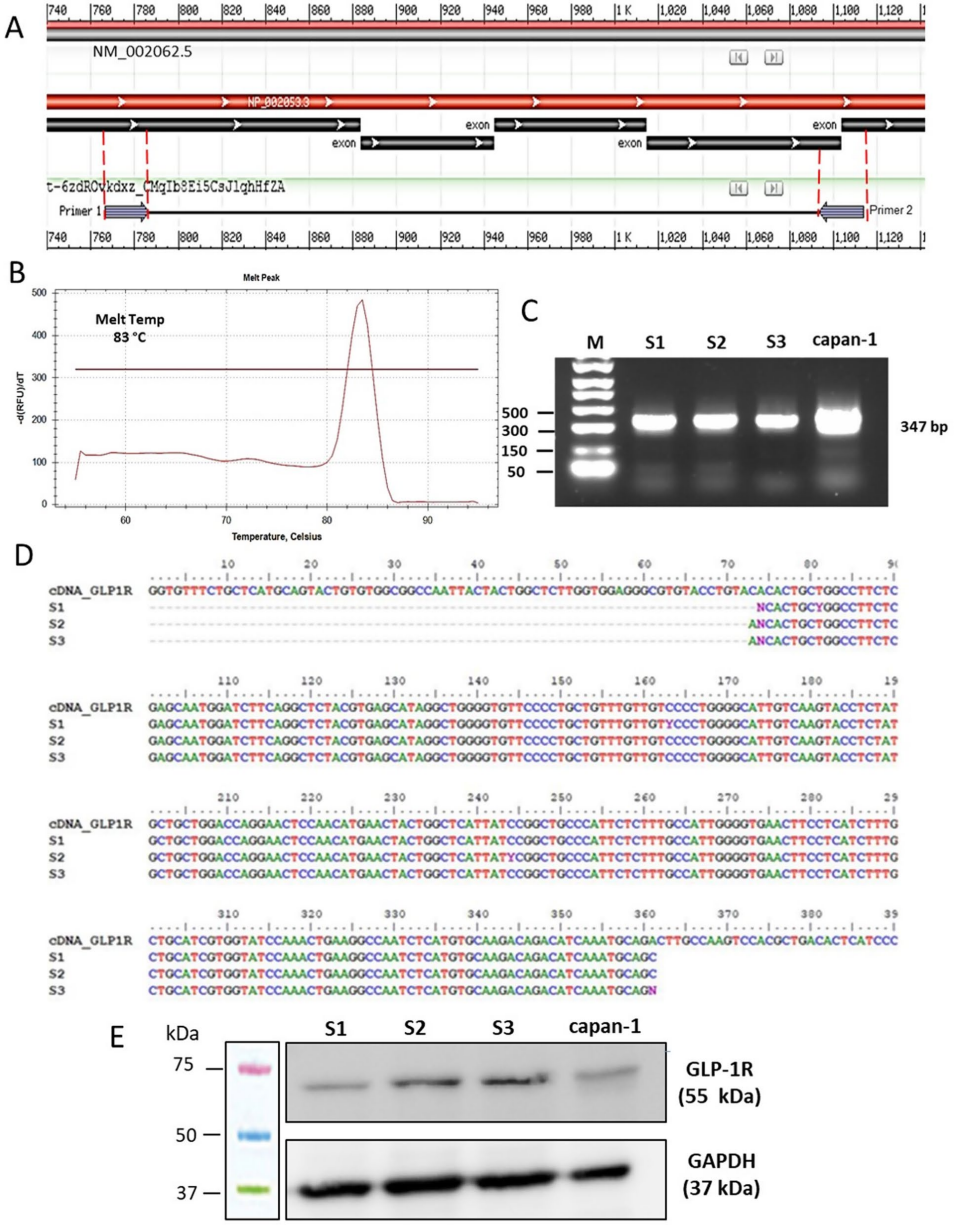
GLP-1R expression in CD34^+^ HSPCs. mRNA expression was assessed by RT-qPCR in 3 different CD34^+^ HSPC biological replicates. An exon-exon spanning reverse primer was used to exclude any amplification of possible contaminating DNA; capan-1 cells were used as positive control (**A**). The identity of RT-qPCR products was determined by melting curve (**B**), agarose gel run (347 bp) (**C**) and finally by Sanger sequencing (**D**). CD34^+^ HSPC lysates (40 μg) deriving from three biological replicates were immunoblotted in order to evaluate GLP-1R protein expression. Capan-1 cell lysate (20 μg) was used as positive control (**E**). S1, S2, S3 = samples 1, 2, 3

We profiled the expression of GLP-1R in CD34^+^ HSPCs at both mRNA and protein levels. In order to ensure cDNA amplification of the target mRNA and exclude PCR products from DNA contamination, we designed a couple of primers spanning exon-exon junction (Fig. 2A). Additionally, to confirm the correct amplification of the target template, the amplicon of 347 bp, characterized by a melting temperature of 83 °C, was resolved on 1% agarose gel. The bands were then excised and sequenced. The alignment of sequenced PCR end-point products with reference coding cDNA (NM_002062.5) showed 99% of identity (Fig. 2B, C and D). Additionally, in order to further support our hypothesis, GLP-1R mRNA expression was confirmed in CD34^+^ HSPCs isolated from sternal BM biopsy of T2DM patients underwent bypass surgery (Additional file 3: Figure S3). Finally, total protein cell lysates obtained from 3 different samples of CD34^+^ HSPCs were subjected to Western blot analysis. Protein cell lysate of capan-1 cells was used as positive control. As shown in Fig. 1E, GLP-1R antibody detected a unique, distinct protein band of the expected molecular weight (55 kDa) in all samples.

### The administration of GLP-1 receptor agonist LIRA stimulates intracellular cAMP production

GLP-1Rs are known to be coupled to activation of Gαs proteins. In pancreatic β cells the receptor agonist engagement results in activation of adenylate cyclase with consequent production of 3',5'-cyclic adenosine monophosphate (cAMP), intracellular Ca^2+^ increase and insulin release [15]. To determine whether CD34^+^ HSPCs express a functional GLP-1R, we assessed intracellular cAMP production and Ca^2+^ mobilization after LIRA stimulation.

**Fig. 3:**
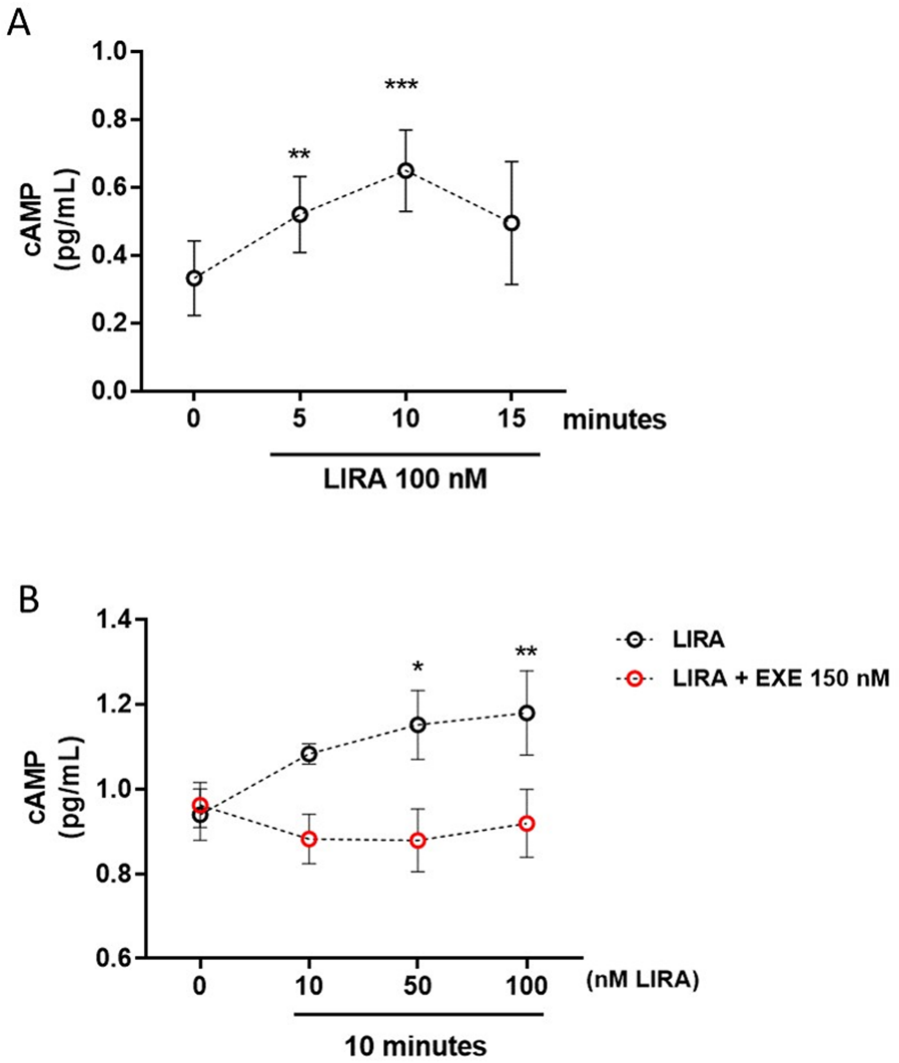
GLP-1R downstream signaling pathway activation after stimulation by LIRA treatment. **A** CD34^+^ HSPC stimulation with 100 nM LIRA determined a significant time-dependent accumulation of intracellular cAMP, with a maximum peak after 10 min of treatment (**p ≤ 0.01; ***p ≤ 0.001 *vs* basal; one-way ANOVA). **B** The addition of 150 nM EXE, a competitive antagonist of GLP-1R prevented the intracellular cAMP accumulation induced by increasing concentration of LIRA (10–100 nM) (*p ≤ 0.05, **p ≤ 0.01 *vs* basal two-way ANOVA). EXE = exendin 9–39, LIRA = liraglutide

Consistent with activation of Gαs, the treatment of cells with LIRA elicited a significant accumulation of intracellular cAMP over basal level in a time- and dose dependent manner reaching the highest value after 10 min of stimulation at 100 nM (Fig. 3A and B). Notably, the addition of the selective GLP-1R antagonist exendin (9-39) (EXE) prevented intracellular cAMP accumulation at all tested LIRA concentrations, demonstrating a receptor-mediated effect (Fig. 3B). Interestingly, single cell analysis showed occurrence of spontaneous Ca^2+^ transients in CD34^+^ HSPCs not significantly altered by 100 nM LIRA addition (Additional file 4: Figure S4A). Moreover, cell population analysis showed negligible increase in intracellular Ca^2+^ following LIRA addition (+ 4% over basal Ca^2+^ level, Additional file 4: Figure S4B). Thus, unlike pancreatic β-cells, acute GLP-1R stimulation in CD34^+^ HSPCs did not significantly alter intracellular Ca^2+^.

### *GLP-1R stimulation prevents CD34*^+^*HSPC dysfunction induced by chronic glucose overload*

We recently published that metabolic stress induced by prolonged HG exposure results in loss of cell proliferation ability and CXCR4/SDF1-α axis impairment [14]. Herein, we tested whether LIRA, a GLP-1RA, was able to avert all these functional damages.

**Fig. 4:**
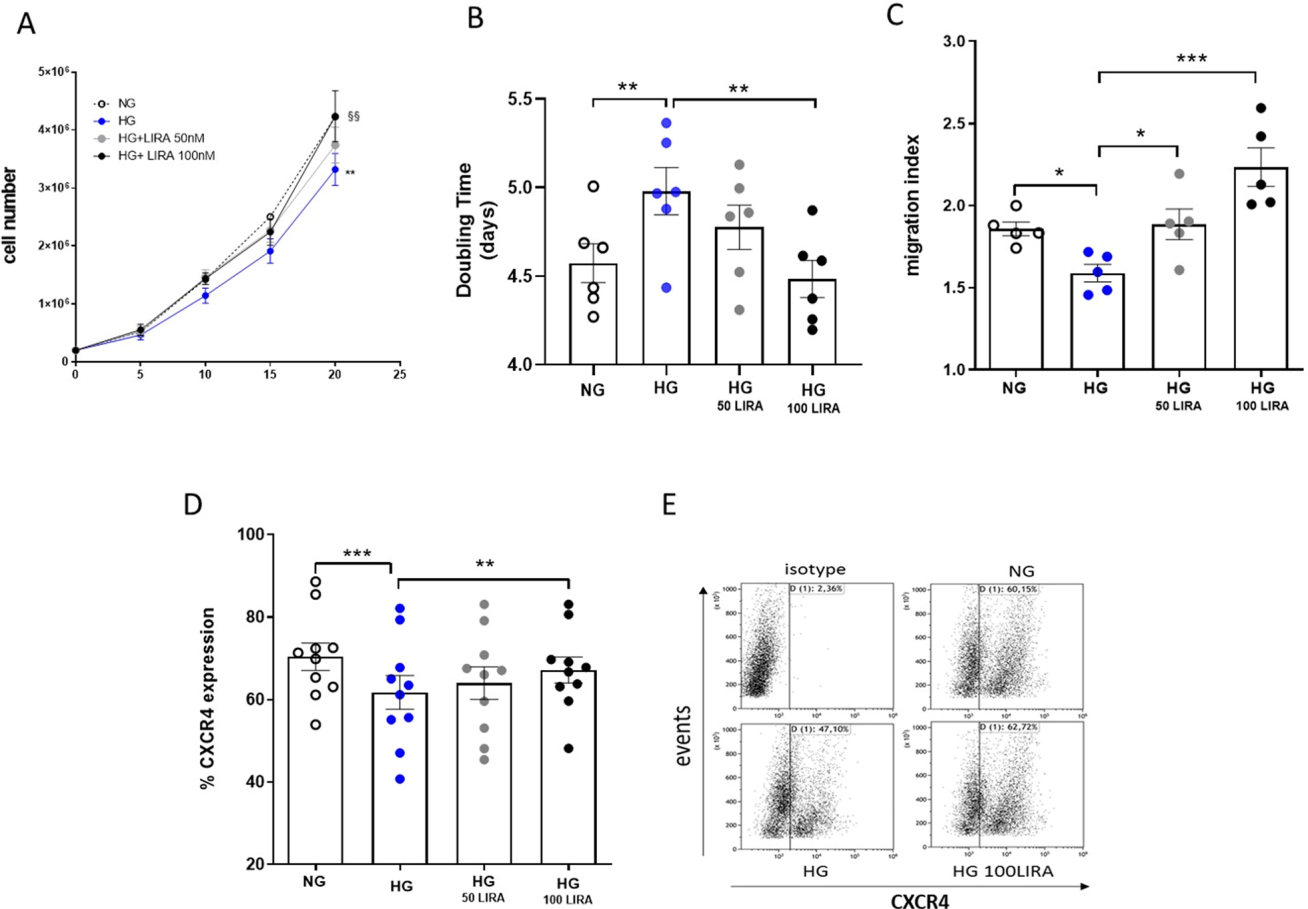
LIRA treatment prevented CD34^+^ HSPC proliferation, CXCR4/SDF-1α axis impairment and ROS accumulation caused by 20 days HG exposure. **A** Proliferation curve of CD34^+^ HSPC amplified in NG, HG ± 50 nM and 100 nM LIRA conditions (**p ≤ 0.01 HG *vs* NG; §§ p ≤ 0.01 HG 100 LIRA *vs* HG; two-way ANOVA). **B** Doubling time of CD34^+^ HSPCs cultured in NG, HG ± 50 nM and 100 nM LIRA (**p ≤ 0.01 HG *vs* NG; **p ≤ 0.01 HG 100 LIRA *vs* HG; one- way ANOVA). **C** CD34^+^ HSPC migration towards SDF-1α (50 ng/mL) after NG, HG ± 50 nM and 100 nM LIRA treatment (*p ≤ 0.05 HG *vs* NG; *p ≤ 0.05 HG 50 LIRA *vs* HG; ***p ≤ 0.001 HG 100 LIRA *vs* HG; one-way ANOVA). **D** Analysis by flow cytometry of CXCR4 expression in NG, HG ± 50 nM and 100 nM LIRA treated CD34^+^ HSPCs (***p ≤ 0.001 HG *vs* NG; ** p ≤ 0.01 HG 100 LIRA *vs* HG; one-way ANOVA). **E** Representative dot plot of CXCR4 cytofluorimetric analysis. HG = high glucose, LIRA = liraglutide, NG = normal glucose

Despite the chronical exposure to HG concentration, LIRA dose-dependently (50 and 100 nM), prevented cell proliferation impairment. Noteworthy, the presence of 100 nM LIRA maintained cell proliferation rate to the control values (NG) (Fig. 4A and B). Similarly, LIRA treatment prevented CXCR4/SDF1-α axis defect promoted by HG exposure. Indeed, LIRA significantly maintained in dose dependent manner the migration ability of cells toward 50 ng/mL of SDF-1α (Fig. 4C) and CXCR4 expression (Fig. 4D and E).

### *LIRA reduces the oxidative state of CD34*^+^*HSPCs and metabolic imbalance promoted by HG exposure*

**Fig. 5:**
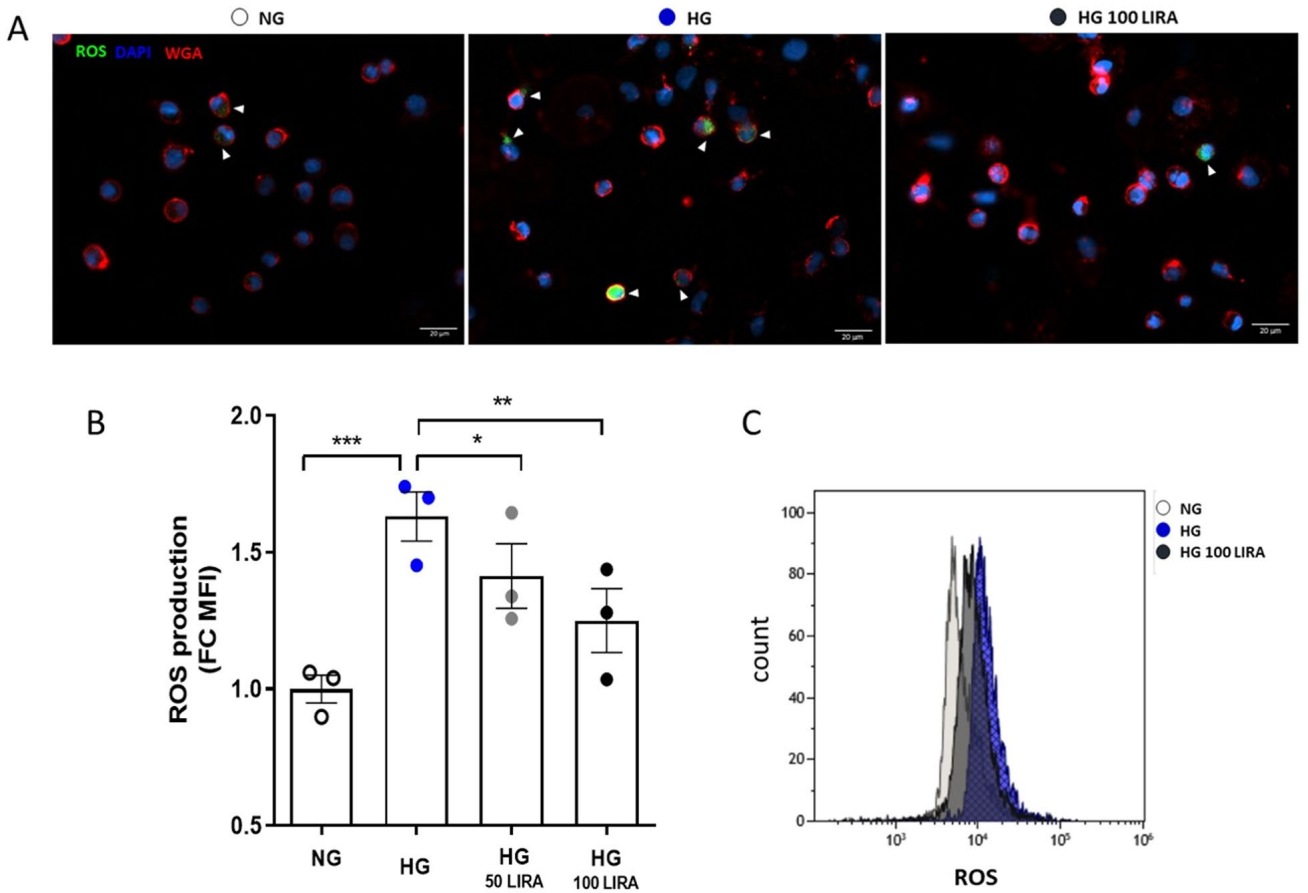
Quantification of intracellular ROS level by immunocytochemistry (A) and flow cytometry (B) in NG, HG ± 50 nM and 100 nM LIRA treated cells (***p ≤ 0.001 HG *vs* NG; *p ≤ 0.05 HG 50 LIRA *vs* HG; ** p ≤ 0.01 HG 100 LIRA *vs* HG; one-way ANOVA). C) Representative flow cytometry histograms of intracellular ROS quantification. HG = high glucose, LIRA = liraglutide, NG = normal glucose

We previously demonstrated that the loss of function promoted by HG-exposure was incident with mitochondrial ROS accumulation [14]. As shown in Fig. 5A and B, the maintenance of functional parameters despite HG presence was associated with a significant drug-induced reduction of cell oxidative state.

**Fig. 6:**
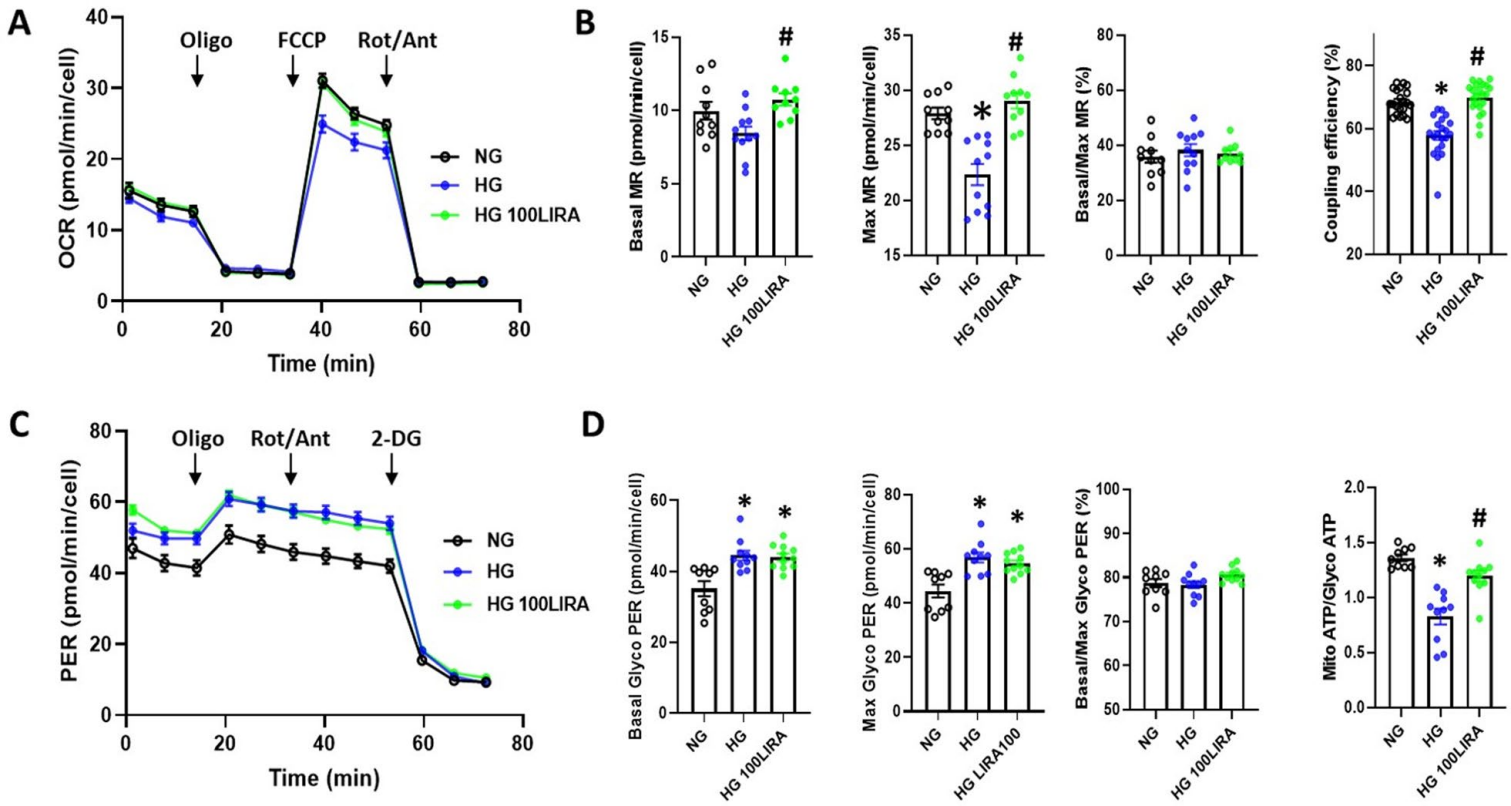
LIRA recovered HG-damaged CD34^+^ HSPC oxidative metabolism. Analysis of bioenergetics profiling with Seahorse in control (NG), after 20 days HG exposure alone (HG) or in the presence of 100 nM LIRA (HG 100LIRA). **A** Oxygen Consumption Rate (OCR) profiles of cells subjected to sequential injections of 1.5 µM oligomycin A, 2 µM FCCP and 0.5 µM rotenone + 0.5 µM antimycin A (XF Mito Stress Test protocol). OCR values were normalized to cell number by counting Hoechst-positive nuclei at the end of the experiment. **B** Statistics of mitochondrial parameters, including basal Mitochondrial Respiration (Basal MR), maximal Mitochondrial Respiration (Max MR), basal to maximal MR ratio and coupling efficiency. **C** Proton Efflux Rate (PER) profiles of cells subjected to sequential injections of 1.5 µM oligomycin A, 0.5 µM rotenone + 0.5 µM antimycin A and 50 mM 2-Deoxy-D-Glucose (combined XF ATP and Glyco rate protocols). **D** Statistics of glycolytic bioenergetic parameters, including basal Glycolysis (Basal GlycoPER), maximal Glycolysis (Max GlycoPER), basal to maximal GlycoPER ratio, ATP rate index (Mito ATP/Glyco ATP). *p < 0.05 vs NG, # p < 0.05 vs HG (one-way ANOVA plus Tukey’s multiple comparison). Bioenergetic parameters were calculated from Seahorse data as described in Methods. Mean ± SEM from two individual experiments (n = 10–24 technical replicates). HG = high glucose, LIRA = liraglutide, NG = normal glucose

Moreover, HG-induced mitochondrial ROS accumulation was associated to oxidative metabolism dysfunction (Fig. 6). HG exposed CD34^+^ HSPCs showed reduced maximal and spare MR, without significantly affecting basal MR. LIRA (100 nM) was able to restore maximal and spare MR to NG levels (Fig. 6A and Additional file 5: Figure S5A). Notably, neither HG-exposure nor LIRA treatment altered the percentage of the mitochondrial machinery used under basal conditions, as indicated by basal to maximal MR ratio, which remained constant in all groups. LIRA also restored the basal respiration devoted to ATP production (here reported as coupling efficiency, Fig. 6A). These effects of both HG and LIRA treatment were correlated to a trend in proton leak alteration (Additional file 5: Figure S5B). From the analysis of the glycolytic parameters, it emerged that HG-exposure increased the basal and maximal glycolysis and ATP produced via this metabolic route (Fig. 6B). Unlike the mitochondrial function, LIRA was unable to restore HG-induced glycolytic bioenergetic parameters changes to NG levels.

Overall, following HG exposure, LIRA treatment improved mitochondrial metabolism, stimulated ATP production via OXPHOS (Additional file 5: Figure S5C and S5D) and thereby increased the ATP rate index (Fig. 6).

### GLP-1R stimulation promotes activation of ERK1/2 and PI3K signaling pathways

**Fig. 7:**
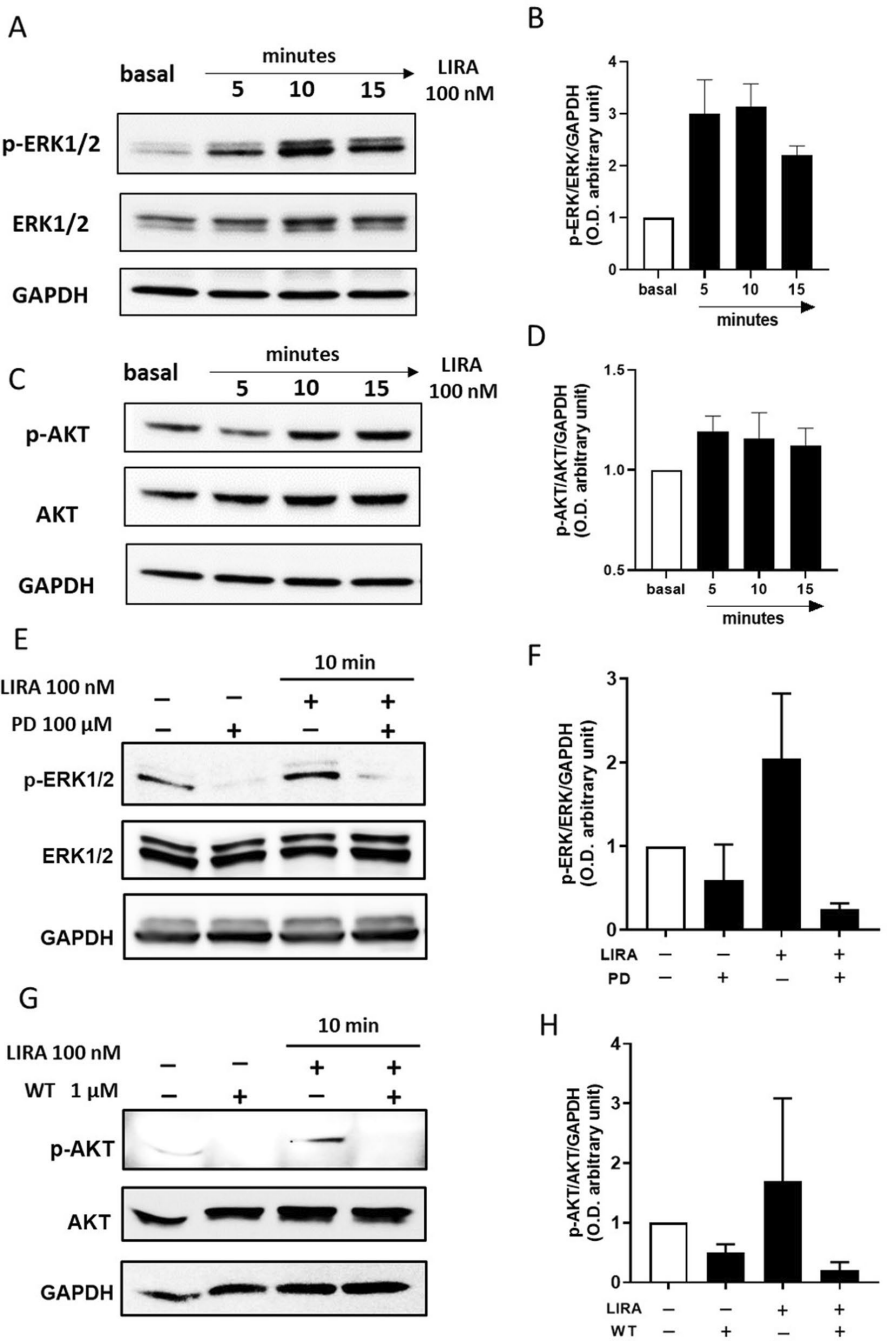
Intracellular pathway cross-talk after GLP-1R1 stimulation with 100 nM LIRA. **A** Representative immunoblots of ERK1/2 and **C** AKT phosphorylation in CD34^+^ HSPCs treated with 100 nM LIRA for 5, 10 and 15 min and after the addition of MEK1/2 and PI3K selective pathway inhibitors, PD (**E**) and WT (**G**) respectively. GAPDH was used as loading control. **B**, **D**, **F**, **H** Immunoblotting quantification presented as arbitrary units after normalization to the GAPDH protein levels of three independent experiments. AKT = protein kinase B, ERK1/2 = extracellular signal-regulated kinases 1 and 2 GAPDH = Glyceraldehyde 3-phosphate dehydrogenase, LIRA = liraglutide, p-AKT = phospho-AKT, p-ERK1/2 = phospho-ERK1/2, PD = PD 98,059, WT = wortmannin

Stimulation of the GLP-1R is known to activate numerous pleiotropic signaling pathways in human pancreatic islet cells including PI3K and extracellular regulated kinases 1 and 2 (ERK1/2) [16, 17]. To determine whether the stimulation of endogenous GLP-1R expressed in CD34^+^ HSPCs was also coupled to similar signal transduction pathways, we assessed the phosphorylation of ERK1/2 and AKT (a downstream effector of PI3K). As shown in Fig. 7A and C, stimulation of the cells with 100 nM LIRA elicited a time-dependent activation of both ERK1/2 and AKT kinases. LIRA-dependent kinase activation was abrogated by the addition of the two selective ERK1/2 and PI3K inhibitors: PD and WT, respectively (Fig. 7E and G).

These data indicate that CD34^+^ HSPCs express a functional GLP-1R whose stimulation is coupled to additional signaling pathways other than adenylate cyclase.

### Exendin (9–39) antagonizes LIRA effects against hyperglycemia

**Fig. 8:**
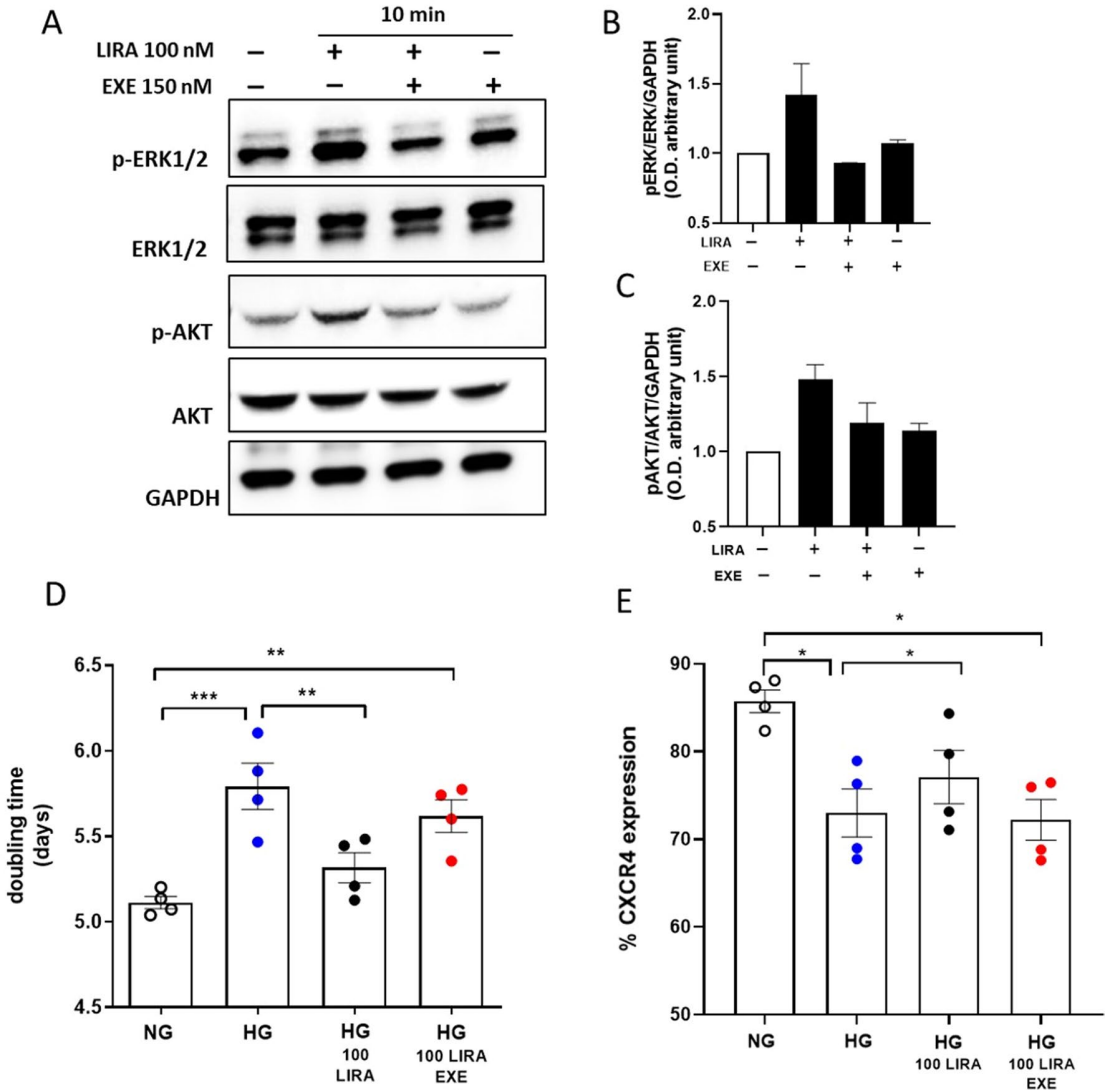
The GLP-1R antagonist EXE abolished ERK1/2 and AKT phosphorylation as well as the protective effects promoted by LIRA treatment. **A** Representative immunoblot of ERK1/2 and AKT phosphorylation after stimulation with LIRA 100 nM ± EXE 150 nM for 10 min. GAPDH was used as loading control. **B**, **C** Immunoblotting results presented as arbitrary units after normalization to the GAPDH protein levels. **D** Doubling time of CD34^+^ HSPCs cultured in NG, HG ± 50 nM and 100 nM ± EXE conditions (***p ≤ 0.001 HG *vs* NG; ** p ≤ 0.01 HG 100 LIRA *vs* HG; ** p ≤ 0.01 HG 100 LIRA + EXE *vs* NG; one-way ANOVA. E) Analysis by flow cytometry of CXCR4 expression in NG, HG ± 50 nM and 100 nM ± EXE CD34^+^ HSPCs (*p ≤ 0.05 HG *vs* NG; *p ≤ 0.05 HG 100 LIRA *vs* HG; *p ≤ 0.05 HG 100 LIRA + EXE *vs* NG; one-way ANOVA). EXE = exendin 9–39, HG = high glucose, LIRA = liraglutide, NG = normal glucose

In order to confirm that LIRA-induced activation of diverse pro-survival signaling pathways was acting through GLP-1R activation, aforementioned experiments were carried out in the presence or absence of EXE antagonist (150 nM) [18]. As shown in Fig. 8A, EXE abrogated LIRA-dependent ERK1/2 and AKT phosphorylation as well as its protective effect on cell proliferation, measured as doubling time, (Fig. 8D) and CXCR4 membrane expression (Fig. 8E). Taken together, these findings support a GLP-1R-mediated effect of LIRA on intracellular pathways and functions of CD34^+^ HSPCs.

### *LIRA partially recovers the functional damage induced by HG exposure in CD34*^+^*HSPCs*

As we recently reported, a strong antioxidant machinery confers to CD34^+^ HSPCs a particular resistance to HG-induced oxidative stress [14]. However, after antioxidant defense exhaustion, irreversible functional alterations take place. Here, in a different experimental setting (Fig. 1B), we aimed at investigating whether LIRA was also able to recover the compromised cell phenotype induced by HG exposure.

**Fig. 9:**
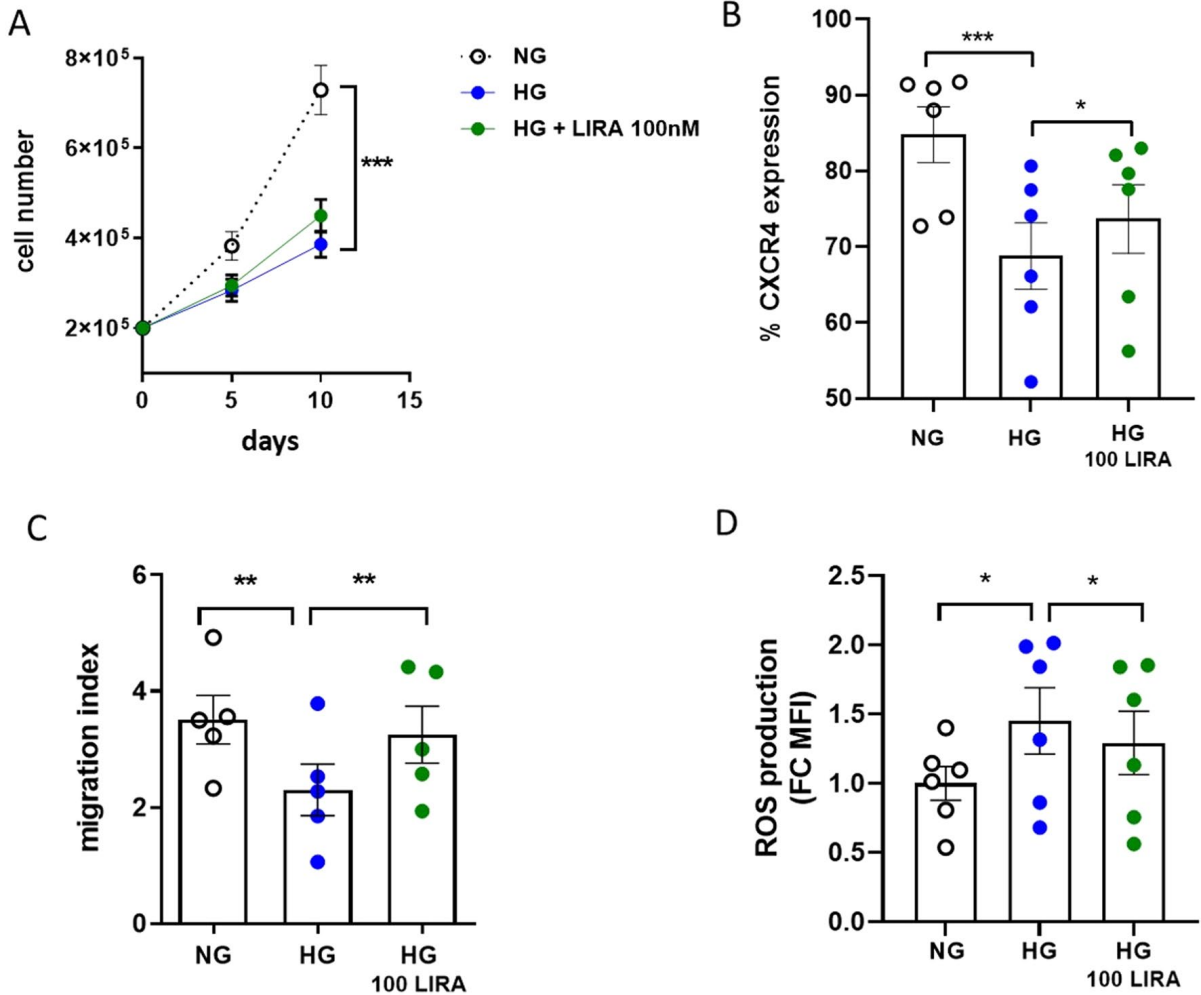
LIRA partially recovered HG-damaged CD34^+^ HSPC functions. **A** Proliferation curve of HG-damaged CD34^+^ HSPCs after 10 days stimulation with 100 nM LIRA (***p ≤ 0.001 HG *vs* NG; two-way ANOVA). **B** Analysis by flow cytometry of CXCR4 expression level in HG-damaged CD34^+^ HSPCs after 10 days of LIRA treatment (***p ≤ 0.001 HG *vs* NG; * p ≤ 0.05 HG 100 LIRA *vs* HG; one-way ANOVA). **C** Migration of HG-damaged CD34^+^ HSPCs after 10 days of LIRA treatment (**p ≤ 0.01 HG *vs* NG; ** p ≤ 0.01 HG 100 LIRA *vs* HG; one-way ANOVA). **D** Quantification of intracellular ROS level by flow cytometry in HG-damaged CD34^+^ HSPCs after 10 days of LIRA treatment (*p ≤ 0.05 HG *vs* NG; * p ≤ 0.05 HG 100 LIRA *vs* HG; one-way ANOVA). HG = high glucose, LIRA = liraglutide, NG = normal glucose

While 100 nM LIRA modestly improved HG-CD34^+^ cell growth rate (Fig. 9A), we found that the same drug concentration significantly recovered CXCR4 membrane expression (Fig. 9B), and restored migration ability of the cells (Fig. 9C), although the latter less efficiently than when added concomitantly to HG. Again, these effects were associated with a significant drug-dependent reduction of intracellular ROS levels (Fig. 9D).

## Discussion

CV complications remain the major cause of morbidity and mortality of patients with DM and first-generation glucose lowering agents have proved to be inadequate [19] or only partially able to favorably impact CV prognosis [20]. Recently, large-scale trials unequivocally demonstrated the CV protective effects of two novel classes of glucose-lowering agents characterized by different dominant mechanism of action: sodium–glucose cotransporter-2 (SGLT2) inhibitors and GLP-1RAs. Both classes of drugs showed to significantly reduce the risk for MACE and all-cause mortality on top of standard of care in T2DM patients [8–10, 21, 22]. These CV benefits, independent from their glucose-lowering action, rely on the enrolment of different and not definitively understood mechanisms at multiple organ systems. Among the numerous pleiotropic actions of GLP-1RAs that favorably affect diabetes comorbidities, the metabolic changes of the patients are determinant. LIRA showed to improve beta cell function, especially in subjects treated with multiple daily insulin injection [23], and to ameliorate circulating metabolome, with particular regard to sphingolipids (e.g. ceramide) [24] and LDL metabolism, this latter by reduction of plasma PCSK9 level [25]. Collectively, these metabolic effects, along with a direct and complementary activity of the drugs at CV level, concur to the beneficial features of GLP-1RAs [26, 27]. Nevertheless, the existence of additional mechanisms has been postulated. CD34^+^ HSPCs are known to play a central role in the maintenance of CV homeostasis by regulating vascular repair and regeneration [28–30]. The importance of CD34^+^ HSPC biological functions on CV outcome [6] is supported by the common ontological origin of vascular and hematopoietic system [31]. Notably, Nandula and colleagues demonstrated that the amelioration of metabolic, CV and renal parameters of T2DM patients after canagliflozin therapy, a SGLT2 inhibitor, was associated with the improvement of CD34^+^ HSPCs function [32]. Although different mechanisms of action are involved in CV protection of SGLT2 inhibitors, this study further supports the hypothesis that part of the mechanisms whereby GLP-1RAs improve CV outcome could rely on LIRA ability to reverse the functional impairment of CD34^+^ HSPC provoked by HG.

### *CD34*^+^*HSPCs express a functional GLP-1R*

We firstly provided unprecedented evidence that cord blood- and BM-derived CD34^+^ HSPCs express GLP-1R (Fig. 2 and Additional file 3: Figure S3). The demonstration of GLP-1R expression by the identification of mRNA transcripts encoding for GLP-1R open reading frame and the use of validated antisera is often lacking and controversial in literature [33]. Therefore, to assess mRNA expression, we designed a couple of primers containing a reverse primer spanning an exon-exon junction. This granted the sole amplification of GLP-1R transcripts excluding any products deriving from possible DNA contamination, as we successfully confirmed by qPCR amplification of genomic DNA (data not shown). Afterwards, qPCR products were sequenced and aligned with the reference coding cDNA (NM_002062.5) confirming their identity. GLP-1R protein expression was finally detected by a top cited and validated antibody [34, 35], whose specificity was further proven in capan-1 cell lysate, a pancreatic cancer cell line expressing GLP-1R [36].

GLP-1R is a member of the secretin family or class B G protein-coupled receptors (GPCRs) [37]. Consistently with its canonical Gs mediated pathway activation, we showed that CD34^+^ HSPCs express a functional GLP-1R. In fact, LIRA elicited a time- and dose-dependent accumulation of intracellular cAMP that was abrogated by the competitive receptor antagonist EXE (Fig. 3). Noteworthy, differently from what reported in pancreatic β-cells, we observed a weak intracellular intracellular Ca^2+^ mobilization, suggesting that, in CD34^+^ HSPCs, Ca^2+^-mediated signaling pathways may not be fundamental for the biological activity of the receptor [38] (Additional file 4: Figure S4).

### *GLP-1R stimulation prevent HG-induced CD34*^+^*HSPC dysfunction and promotes mitochondrial metabolism*

In the last years, a number of preclinical and clinical studies have demonstrated that native GLP-1 as well as GLP-1 RAs exert pleiotropic effects on different tissue subsets through both GLP-1R-dependent and independent mechanisms [27, 39]. At CV system level, they showed to improve endothelial function, reduce atherosclerosis, as well as oxidative stress and vascular and cardiac inflammation [27]. After GLP-1R expression in HSPC was confirmed, we were puzzled to evaluate its biological effects in a diabetic environment. We recently reported that the loss of glucose tolerance in CD34^+^ HSPCs was associated with the reduction of proliferation rate, increase in mitochondrial ROS production and CXCR4/SDF-1α axis impairment [14]. These functional deficits are known to be primarily involved in the impairment of CD34^+^ HSPC mobilization and migration capacity from the BM to sites of ischemia and endothelial injury in diabetic patients [40, 41]. Here we found that the concomitant administration of LIRA in HG setting was able to prevent HG-induced CD34^+^ HSPC dysfunction and to improve their oxidative state (Figs. 4 and 5). It is well known that HG-induced overproduction of ROS can disrupt the mitochondrial membrane potential and damage mitochondrial function [42–45]. Seahorse analysis confirmed that exposure to HG of CD34^+^ HSPCs induces a significant reduction of ATP produced by mitochondrial respiration (OXPHOS), as well as a reduced maximal and spare respiratory capacity. HG-exposed cells compensate for the impairment of mitochondrial function with an improvement of glycolytic ATP production, associated with increased basal and maximal glycolytic capacity. GLP-1R stimulation by LIRA affected cellular bioenergetics and restored mitochondrial function. These results further support the hypothesis that GLP-1RAs are able to reverse the functional impairment of CD34^+^ HSPC provoked by HG.

Interestingly, in line with recent findings, the protective effects of LIRA persisted throughout the entire duration of the experiment, suggesting a sustained endosomal cyclic AMP generation induced by internalized activated receptor complex [46, 47].

### GLP-1R stimulation promotes the activation of cytoprotective pathways

GLP-1R stimulation is known to promote transactivation of multiple intracellular pathways including PI3K, and ERK1/2. These pathways, which exert proliferative and cytoprotective functions [48], have been described in numerous extra glucose-lowering actions of incretins [39, 49]. In our hands, the treatment with 100 nM LIRA promoted in CD34^+^ HSPCs a time dependent ERK1/2 and AKT phosphorylation that was completely abolished by the addition of PD 98059 and wortmannin, selective MEK1/2 and PI3K inhibitors respectively, and by the co-treatment with the GLP-1R antagonist EXE along with the related protective effects (Figs. 7 and 8). Albeit we are aware that we have not provided a full demonstration of the exact mechanism by which GLP-1R activation mediates ERK1/2 and AKT phosphorylation, we think we provided enough evidence that the protective effect of LIRA in CD34^+^ HSPCs is mediated by GLP-1R, even if other mechanisms cannot be excluded.

According to guidelines, GLP1-RAs are now recommended to reduce the risk of CV events and mortality in T2DM patients. According to our hypothesis, this implies that LIRA cardiovascular protective effects are exerted after CD34^+^ HSPC dysfunction has taken hold. To assess the ability of LIRA to recover a HG-related compromised stem cell phenotype, the drug was given after the dysfunctional phenotype emerged. LIRA was able to recover CD34^+^ HSPC function, even if less efficiently than early administration (Fig. 9). This observation corroborates accumulating evidence for supporting the use of GLP-1 RAs for CVD prevention in T2DM patients.

## Conclusion

We provided first evidence that CD34^+^ HSPCs express GLP-1R, and that the GLP-1RA LIRA prevents proliferation and migration impairment induced by chronic HG exposure. LIRA was also able to improve, even if less efficiently, CD34^+^ HSPC function when previously exposed to HG conditions. Taken together these data suggest that the reported CV benefits of GLP-RAs can at least in part be related to cytoprotective effects on CD34^+^ HSPCs.

## Supplementary Information

**Additional file 1: Figure S1** Flow-cytometric evaluation of sorted CD34^+^ cell purity vs CD14 and CD3 cell contamination.

**Additional file 2: Figure S2** **A** FCCP-uncoupled respiration (Maximal MR) and **B** coupling efficiency calculated from a Mito stress test assay performed on CD34^+^ HSPC grown in NG, HG and HG 100 LIRA using different combinations of oligomycin A (0.5 and 1.5 µM) and FCCP (0.5, 1, 2 and 5 µM) to identify their minimal dose with the maximal response. Data were normalized to 0.5 µM oligomycin A + 0.5 µM FCCP to highlight differences. Mean ± SEM from one experiment (n = 4 technical replicates). Bars indicate statistical differences between groups (one-way ANOVA).

**Additional file 3: Figure S3** GLP-1R expression in BM-derived CD34^+^ HSPCs of T2DM patients. mRNA expression was assessed by RT-qPCR in 3 different biological replicates. The identity of RT-qPCR products was determined by agarose gel run (347 bp). M = marker; PZ1, PZ2, PZ3 = patient 1, 2, 3; B = blank.

**Additional file 4: Figure S4** Intracellular Ca^2+^ mobilization in CD34^+^ HSPCs w/wo GLP-1R stimulation. **A** Spontaneous Ca^2+^ transients elicited in single FLUO4 loaded cells before and during LIRA treatment. **B** Mean FLUO4 fluorescence before and after LIRA injection from a population of CD34^+^ HSPCs (N = 8).

**Additional file 5: Figure S5** Statistics of Spare Mitochondrial Respiration (**A**, Spare MR), Proton Leak (**B**), Mito ATP (**C**) and Glyco ATP (**D**) from ATP rate assay in all experimental groups. Mean ± SEM from two individual experiments (n = 10–24 technical replicates). *p < 0.05 vs NG, # p < 0.05 vs HG (one-way ANOVA plus Tukey’s multiple comparison).

## Abbreviations

AKT/PKB: Protein kinase B
cAMP: Cyclic adenosine monophosphate
CB: Cord blood
CVD: Cardiovascular disease
ECAR: Extracellular acidification rate
ERK1/2: Extracellular signal-regulated kinases 1/2
EXE: Exendin fragment (9-39)
FLT3: Fms‐like tyrosine kinase 3
GLP-1: Glucagon-like peptide 1
GLP1-RAs: GLP-1 receptor agonists
HG: Hyperglycemic
IL: Interleukin
LIRA: Liraglutide
MACE: Major cardiovascular events
MEK1/2: Mitogen-activated protein kinase kinase
MR: Mitochondrial respiration
NG: Normoglycemic
OCR: Oxygen consumption rate
OXPHOS: Oxidative phosphorylation
PER: Proton efflux rate
PI3K: Phosphatidylinositol 3-kinase
PD: PD98059
PVDF: Polyvinylidene difluoride
ROS: Reactive oxygen species
SCF: Stem cell factor
SGLT2: Sodium–glucose cotransporter-2
T2DM: Type 2 diabetes mellitus
WT: Wortmannin
