# Supplementary figures and images for: Liraglutide preserves CD34+ stem cells from dysfunction Induced by high glucose exposure
Source: Cardiovasc Diabetol. 2022 Apr 9;21:51. doi: 10.1186/s12933-022-01486-9 (PMC8994898; doi:10.1186/s12933-022-01486-9)

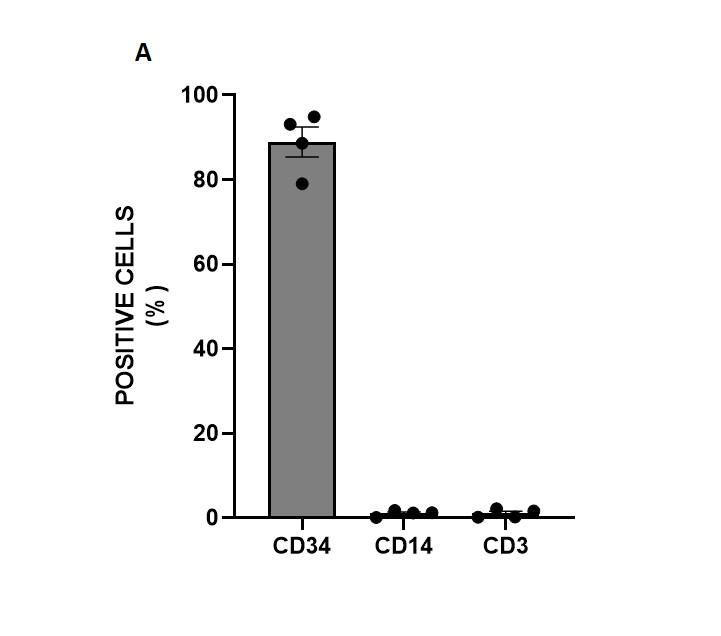

Supplement: Supplementary file 1 — Additional file 1: Figure S1 Flow-cytometric evaluation of sorted CD34+ cell purity vs CD14 and CD3 cell contamination. [file 12933_2022_1486_MOESM1_ESM.tif]

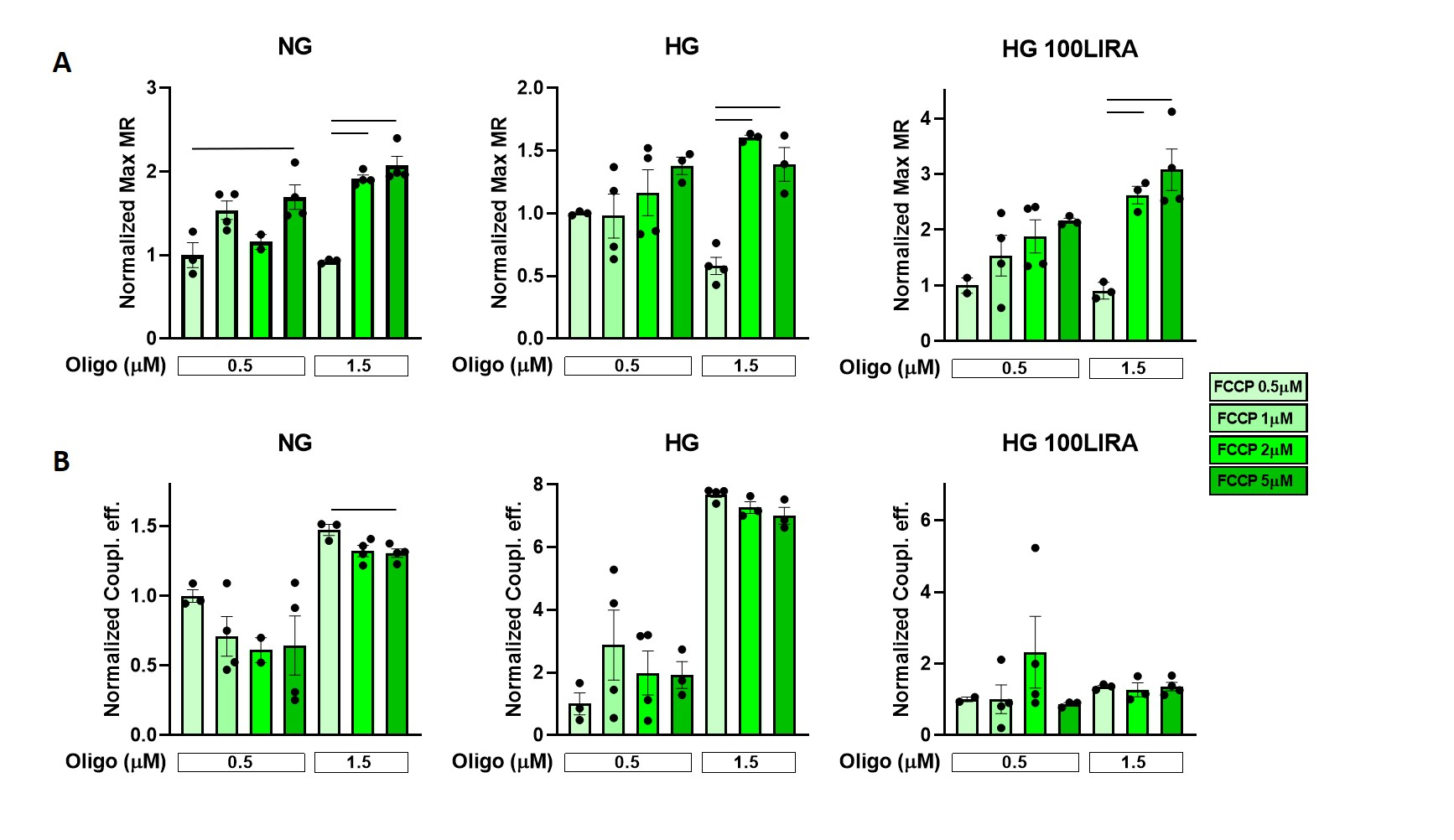

Supplement: Supplementary file 2 — Additional file 2: Figure S2 A FCCP-uncoupled respiration (Maximal MR) and B coupling efficiency calculated from a Mito stress test assay performed on CD34+ HSPC grown in NG, HG and HG 100 LIRA using different combinations of oligomycin A (0.5 and 1.5 µM) and FCCP (0.5, 1, 2 and 5 µM) to identify their minimal dose with the maximal response. Data were normalized to 0.5 µM oligomycin A + 0.5 µM FCCP to highlight differences. Mean ± SEM from one experiment (n = 4 technical replicates). Bars indicate statistical differences between groups (one-way ANOVA). [file 12933_2022_1486_MOESM2_ESM.tif]

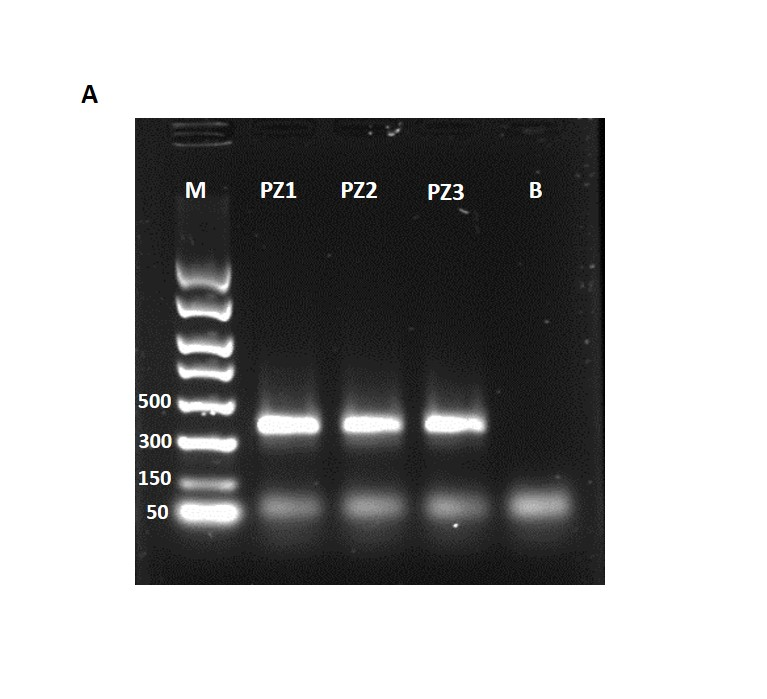

Supplement: Supplementary file 3 — Additional file 3: Figure S3 GLP-1R expression in BM-derived CD34+ HSPCs of T2DM patients. mRNA expression was assessed by RT-qPCR in 3 different biological replicates. The identity of RT-qPCR products was determined by agarose gel run (347 bp). M = marker; PZ1, PZ2, PZ3 = patient 1, 2, 3; B = blank. [file 12933_2022_1486_MOESM3_ESM.tif]

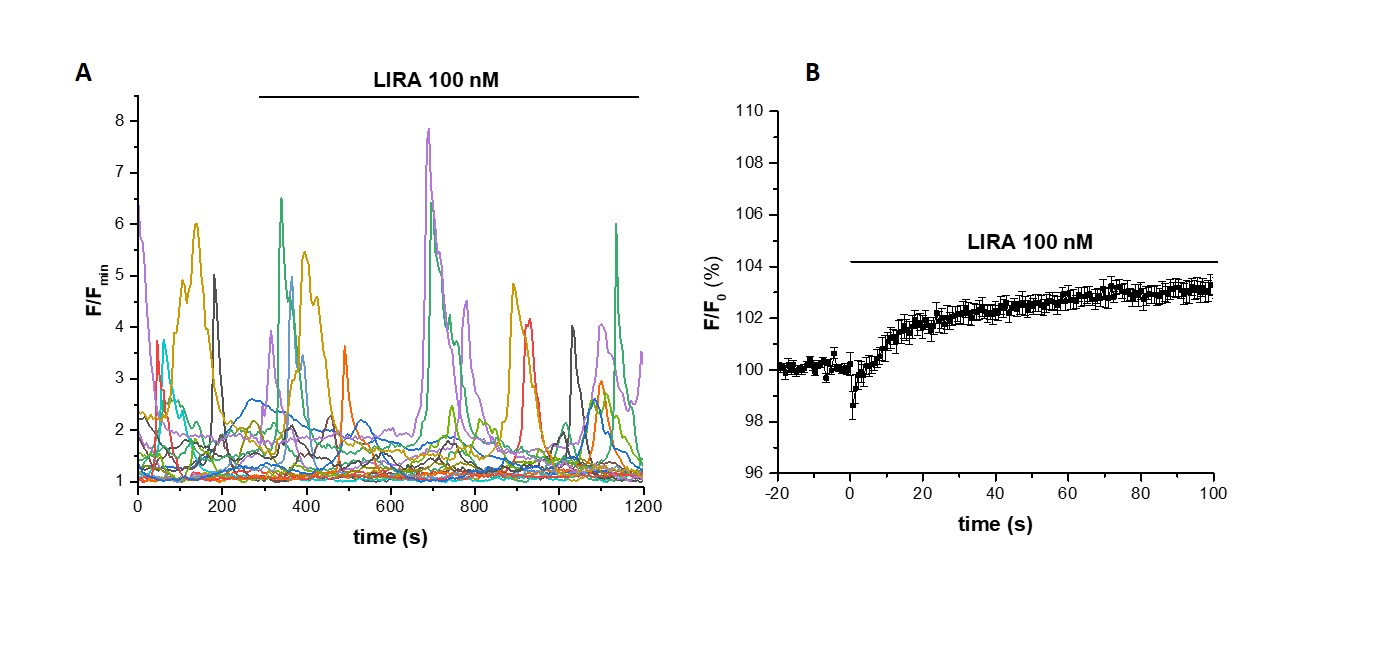

Supplement: Supplementary file 4 — Additional file 4: Figure S4 Intracellular Ca2+ mobilization in CD34+ HSPCs w/wo GLP-1R stimulation. A Spontaneous Ca2+ transients elicited in single FLUO4 loaded cells before and during LIRA treatment. B Mean FLUO4 fluorescence before and after LIRA injection from a population of CD34+ HSPCs (N = 8). [file 12933_2022_1486_MOESM4_ESM.tif]

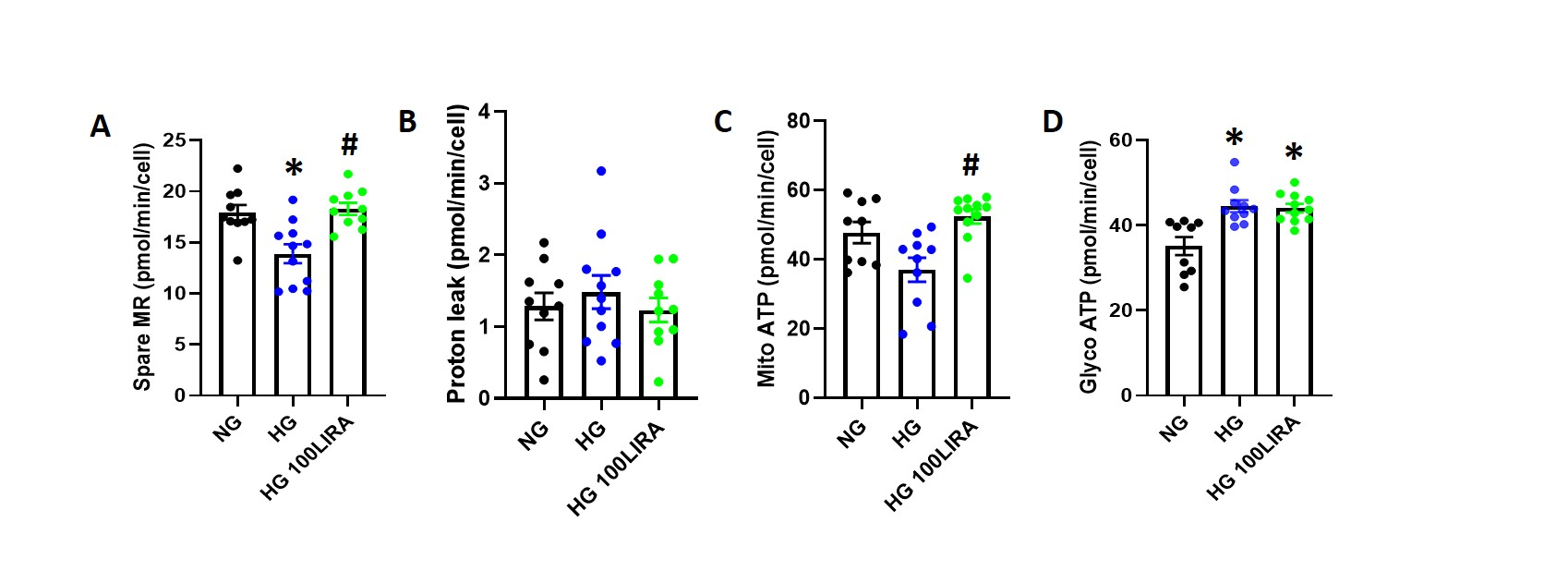

Supplement: Supplementary file 5 — Additional file 5: Figure S5 Statistics of Spare Mitochondrial Respiration (A, Spare MR), Proton Leak (B), Mito ATP (C) and Glyco ATP (D) from ATP rate assay in all experimental groups. Mean ± SEM from two individual experiments (n = 10–24 technical replicates). *p < 0.05 vs NG, # p < 0.05 vs HG (one-way ANOVA plus Tukey’s multiple comparison). [file 12933_2022_1486_MOESM5_ESM.tif]
